# An interactional network of genes involved in chitin synthesis in *Saccharomyces cerevisiae*

**DOI:** 10.1186/1471-2156-6-8

**Published:** 2005-02-16

**Authors:** Guillaume Lesage, Jesse Shapiro, Charles A Specht, Anne-Marie Sdicu, Patrice Ménard, Shamiza Hussein, Amy Hin Yan Tong, Charles Boone, Howard Bussey

**Affiliations:** 1 Department of Biology, McGill University, Montréal (PQ) H3A 1B1, Canada; 2 Department of Medicine, Boston University, Boston MA 02118, USA; 3 Banting and Best Department of Medical Research, University of Toronto, Toronto (ON) M5G 1L6, Canada

## Abstract

**Background:**

In *S. cerevisiae *the β-1,4-linked N-acetylglucosamine polymer, chitin, is synthesized by a family of 3 specialized but interacting chitin synthases encoded by *CHS1*, *CHS2 *and *CHS3*. Chs2p makes chitin in the primary septum, while Chs3p makes chitin in the lateral cell wall and in the bud neck, and can partially compensate for the lack of Chs2p. Chs3p requires a pathway of Bni4p, Chs4p, Chs5p, Chs6p and Chs7p for its localization and activity. Chs1p is thought to have a septum repair function after cell separation. To further explore interactions in the chitin synthase family and to find processes buffering chitin synthesis, we compiled a genetic interaction network of genes showing synthetic interactions with *CHS1*, *CHS3 *and genes involved in Chs3p localization and function and made a phenotypic analysis of their mutants.

**Results:**

Using deletion mutants in *CHS1*, *CHS3*, *CHS4*, *CHS5*, *CHS6*, *CHS7 *and *BNI4 *in a synthetic genetic array analysis we assembled a network of 316 interactions among 163 genes. The interaction network with *CHS3*, *CHS4*, *CHS5*, *CHS6*, *CHS7 *or *BNI4 *forms a dense neighborhood, with many genes functioning in cell wall assembly or polarized secretion. Chitin levels were altered in 54 of the mutants in individually deleted genes, indicating a functional relationship between them and chitin synthesis. 32 of these mutants triggered the chitin stress response, with elevated chitin levels and a dependence on *CHS3*. A large fraction of the *CHS1*-interaction set was distinct from that of the *CHS3 *network, indicating broad roles for Chs1p in buffering both Chs2p function and more global cell wall robustness.

**Conclusion:**

Based on their interaction patterns and chitin levels we group interacting mutants into functional categories. Genes interacting with *CHS3 *are involved in the amelioration of cell wall defects and in septum or bud neck chitin synthesis, and we newly assign a number of genes to these functions. Our genetic analysis of genes not interacting with *CHS3 *indicate expanded roles for Chs4p, Chs5p and Chs6p in secretory protein trafficking and of Bni4p in bud neck organization.

## Background

In vegetatively growing cells of *Saccharomyces cerevisiae*, chitin, a linear polymer of β-1,4-linked N-acetylglucosamine (GlcNAc) residues, is selectively concentrated at the bud neck and is also found as a minor component of the mature lateral cell wall. Chitin is also the main constituent of the primary septum, a structure that separates mother and daughter cells (for reviews, see [1-3]).

Polymerization of UDP-GlcNAc to chitin is catalyzed by a family of three membrane-associated chitin synthases (CS) with specialized activities. CSIII, encoded by *CHS3*, is responsible for synthesis of the chitin ring at the bud neck and for chitin in the lateral wall. CSII synthesizes the chitin of the primary septum, and is encoded by *CHS2*, a gene that is essential in many strain backgrounds [4]. CSI, encoded by *CHS1*, is localized to the plasma membrane and to chitosome vesicles [5] and mutants are hypersensitive to the chitin synthase inhibitor, polyoxyin D, and under acid conditions can form small aberrant buds that are prone to lysis [6]. Disruption of the chitinase gene *CTS1 *required for cell separation suppresses the *chs1 *lysis phenotype, leading to the suggestion that Chs1p is involved in chitin repair at cytokinesis [7].

The precise deposition of chitin is achieved through spatial and temporal controls on each chitin synthase which determine their localization and activity. CSII is expressed in a cell cycle-dependent manner, and is transported to the bud neck through the secretory pathway, and subsequently degraded in the vacuole [8,9]. CSI and III are transported to a specialized endosome-derived compartment, the chitosome, from which they are mobilized by regulated secretion to the plasma membrane [5,8,10]. The localization and trafficking of Chs3p require *BNI4*, *CHS4/SKT5*, *CHS5*, *CHS6 *and *CHS7*. Chs7p is required for exit of Chs3p from the endoplasmic reticulum [11], while Chs5p and Chs6p are involved in transport of Chs3p from the chitosome to the plasma membrane [12,13]. Chs3p forms a complex with Chs4p/Skt5p, a protein required for Chs3p activity during vegetative growth, and Bni4p localizes this complex to the septin ring at the bud neck [14].

Although accounting for only 1–2% of the wild type cell wall under vegetative growth, chitin can contribute up to 20% of the cell wall under the conditions of cell wall stress found in cell wall mutants or on drug exposure [3]. Indeed, in response to cell wall stress Chs3p activity is up-regulated leading to an increased synthesis of chitin, which can be essential for survival. For instance, *CHS3 *is essential for maintaining the cell integrity of several cell wall mutants, such as *fks1 *or *gas1 *[15-17]. Similarly, defective primary septum synthesis can be compensated for by Chs3p-dependent formation of a remedial septum, resulting in a synthetic lethal interaction between *CHS2 *and *CHS3 *[4].

To further explore the relationship between chitin synthesis and other pathways, we assemble a network of 316 synthetic interactions of 163 genes with genes involved in the regulation of chitin synthesis. The relationship of these genes with chitin synthesis was analyzed by measuring the chitin content of the 156 viable deletion mutants and by testing for Calcofluor white sensitivity phenotypes of the 116 deletion mutants in non-essential genes of the CSIII network.

## Results

### A network of genetic interactions with genes involved in chitin synthase function

**Figure 1:**
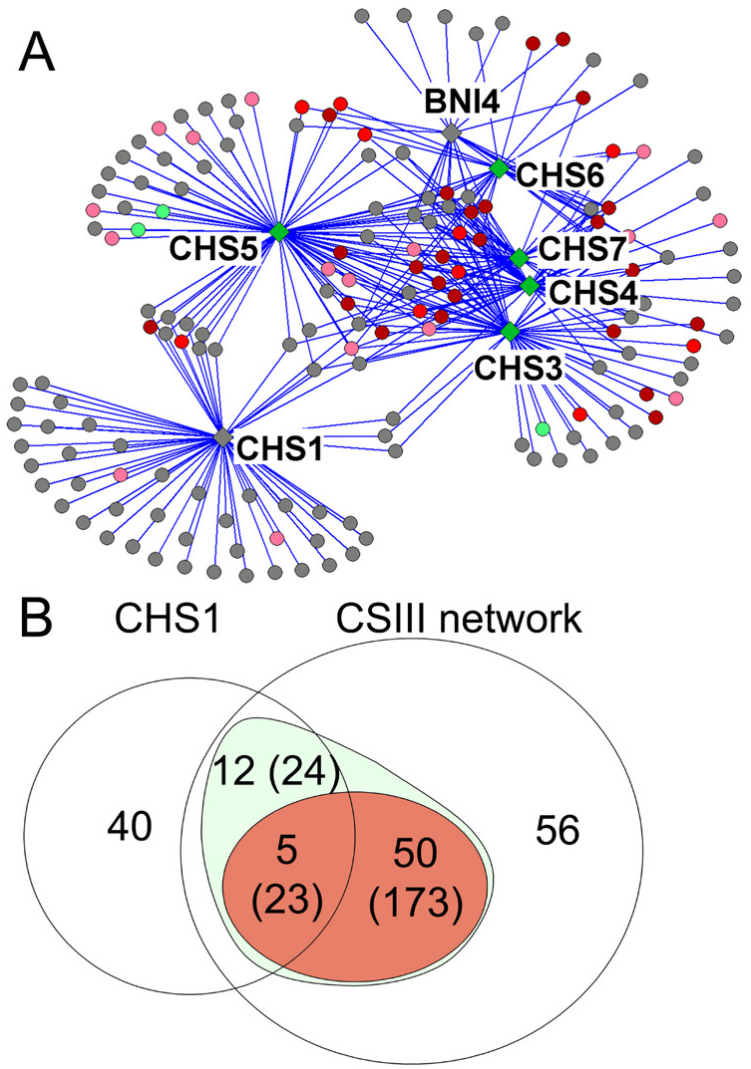
**A network of genetic interactions with *BNI4*, *CHS1*, *CHS3*, *CHS4*, *CHS5*, *CHS6 *and *CHS7***. (A) Global view of the network. Synthetic interactions with any query gene (diamonds) are depicted as edges joining these to nodes (circles). Nodes whose deletion mutant have a decreased, wild type and increased chitin content are colored in green, gray and red, respectively. For the decreased (green) and increased (red) chitin contents, color intensity is proportional to the magnitude of the change. (B) Venn diagram of the *CHS1 *interaction set with the CSIII network. The number of genes interacting with *CHS1 *or with any of the CSIII query genes is indicated. The numbers in parentheses indicate the number of interactions for multiply connected genes. Genes showing 2 or more interactions are grouped in green or red ovals, respectively.

To identify genes buffering defects in chitin synthesis, we searched for genes engaged in synthetic interactions with *BNI4*, *CHS1*, *CHS3*, *CHS4*, *CHS5*, *CHS6 *or *CHS7 *using the SGA methodology [18,19]. Our results identified 163 genes involved in 316 synthetic interactions that form a network in which *BNI4*, *CHS1*, *CHS3*, *CHS4*, *CHS5*, *CHS6 *and *CHS7 *are connected to 22, 57, 63, 47, 71, 25 and 31 genes, respectively (Table 2). Genes interacting with *BNI4, CHS3, CHS4, CHS5, CHS6 *or *CHS7 *tend to be multiply connected, while those interacting with *CHS1 *form a more distinct subnetwork (Figure 1A). Indeed, just 17 of the 57 *CHS1 *interacting genes show an additional interaction with at least another query gene (Figure 1B). In contrast, 67/123 genes interacting with *BNI4 *or *CHS3-7 *are multiply connected (Figure 1B, green oval), and 55 of those show an interaction with either *BNI4 *or *CHS3-7 *(Figure 1B, red oval) resulting in a densely connected CSIII network.

**Table 2:** Synthetic interactions with *BNI4*, *CHS1*, *CHS3*, *CHS4*, *CHS5*, *CHS6 *and *CHS7*.

**Functional category**	**Gene**	**Interacting partners**
Cell wall maintenance	*BCK1*	*BNI4, CHS1, CHS3, CHS4, CHS5, CHS7*
	*FKS1, SLT2, SMI1*	*BNI4, CHS3, CHS4, CHS5, CHS6, CHS7*
	*YPL261C*	*CHS1*
	*ECM21*	*CHS1, CHS5*
	*CHS2*	*CHS3*
	*SWI4*	*CHS3, CHS4, CHS5*
	*CCW12, GAS1, YLR111W*	*CHS3, CHS4, CHS5, CHS7*
	*TUS1*	*CHS4, CHS5, CHS7*
	*DAN3, PAT1*	*CHS5*
Cell polarity & vesicular transport	*NBP2, RGD1, SHS1, SPA2*	*BNI4*
	*EDE1, MYO2, RVS167, VRP1*	*BNI4, CHS3, CHS4, CHS5, CHS7*
	*ARC40, ARP2*	*BNI4, CHS3, CHS4, CHS5, CHS6, CHS7*
	*BNI1*	*BNI4, CHS3, CHS4, CHS7*
	*RVS161*	*BNI4, CHS5*
	*CYK3*	*BNI4, CHS7*
	*BUD20, VPS5, VPS17, VPS29, VPS35*	*CHS1*
	*HBT1*	*CHS1, CHS3*
	*ARC18*	*CHS1, CHS3, CHS4, CHS5, CHS6*
	*EMP24*	*CHS1, CHS3*
	*BEM4, PEA2*	*CHS1, CHS5*
	*CDC3, CDC11, IES6, SRV2, VAM7*	*CHS3*
	*CDC12*	*CHS3, CHS4*
	*FAB1*	*CHS3, CHS4, CHS5*
	*CLA4*	*CHS3, CHS4, CHS5, CHS6, CHS7*
	*SAC6, SLA1, TPM1*	*CHS3, CHS4, CHS5, CHS7*
	*YLR338W*	*CHS3, CHS4, CHS6*
	*SHE4, SMY1*	*CHS3, CHS4, CHS7*
	*VPS24, VPS67*	*CHS3, CHS5*
	*AST1, LST4, YPK1*	*CHS4*
	*SEC22*	*CHS4, CHS5*
	*AOR1, HSE1, VPS21*	*CHS5*
Suspected role in cell polarity & vesicular transport	*YPL066W*	*BNI4*
	*ILM1*	*BNI4, CHS3, CHS4, CHS6*
	*SPF1*	*CHS1, CHS4*
	*YGL081W*	*CHS1, CHS5*
	*YBR077C*	*CHS3*
	*GUP1*	*CHS3, CHS4, CHS5, CHS6*
	*OPI3*	*CHS3, CHS4, CHS7*
	*LSM6*	*CHS5*
	*IST3*	*CHS6*
Protein modification	*VAN1*	*BNI4, CHS3, CHS4, CHS5, CHS6, CHS7*
	*YGL110C*	*CHS1*
	*ANP1, BTS1, MNN2, MNN9*	*CHS3*
	*MNN10*	*CHS3, CHS4, CHS5, CHS6, CHS7*
	*UBI4*	*CHS3, CHS5, CHS6*
	*UBP13*	*CHS4*
	*BRE1*	*CHS5*
	*UFD4*	*CHS5*
	*MNN11*	*CHS5, CHS6*
	*LAS21*	*CHS6*
Ribosomal function/cell size	*LGE1*	*CHS1, CHS3, CHS5*
	*RPL20B*	*CHS3*
	*RPS8A*	*CHS3, CHS4, CHS5*
	*ASC1*	*CHS3, CHS5*
	*RSA1*	*CHS4*
	*RPL14A*	*CHS5*
Cell cycle	*CLN2*	*CHS1*
	*CDC26, DOC1*	*CHS3*
	*YNL171C*	*CHS3, CHS5*
	*CLB3, CTK2*	*CHS5*
Mitochondrial function	*MDM38, NUC1, UTH1, YME1*	*CHS1*
	*YFR045W*	*CHS1, CHS5*
	*MST1, TOM37*	*CHS3*
	*YTA12*	*CHS3, CHS4*
	*ATP17*	*CHS4*
	*RPO41*	*CHS4, CHS6, CHS7*
	*COQ2, COX11, LAT1, MDM12, PET8, SHE9*	*CHS5*
Carbohydrate and lipid metabolism	*DEP1, ELO1, HXT8, IPK1, PDA1, PDC1, PFK2, PHO5, PKR1, RPE1, TYR1, YDR248C*	*CHS1*
Other functions	*BRE5*	*BNI4, CHS3, CHS4*
	*IXR1*	*BNI4, CHS5*
	*CNB1, HAP2, HIT1, PEX22, PMP3, PRM3, SKI2, WHI2*	*CHS1*
	*RPA34*	*CHS1, CHS3, CHS4, CHS5, CHS7*
	*FPS1*	*CHS1, CHS4, CHS5*
	*GRS1, LEA1, MRE11*	*CHS1, CHS5*
	*CSF1*	*CHS3, CHS4, CHS5*
	*PRE9*	*CHS3, CHS5, CHS7*
	*MUM2*	*CHS3, CHS6*
	*UME6*	*CHS4, CHS6*
	*DOT1, PDE2, PEX14, SWI3*	*CHS5*
	*IRA2*	*CHS5, CHS6*
	*NUP133*	*CHS5, CHS6, CHS7*
	*MSN5*	*CHS6*
	*PEX6*	*CHS7*
Unknown function	*YBR209W, YDR314C, YEL033W, YIL110W, YMR003W, YNL179C, YOR322C, YPR053C*	*CHS1*
	*YDL206W*	*CHS1, CHS5*
	*YDL032W*	*CHS3*
	*YDL033C*	*CHS3, CHS5*
	*YGL152C*	*CHS5*
	*YNL235C*	*CHS6*
	*YIL121W*	*CHS7*

### The CSIII network

**Figure 2:**
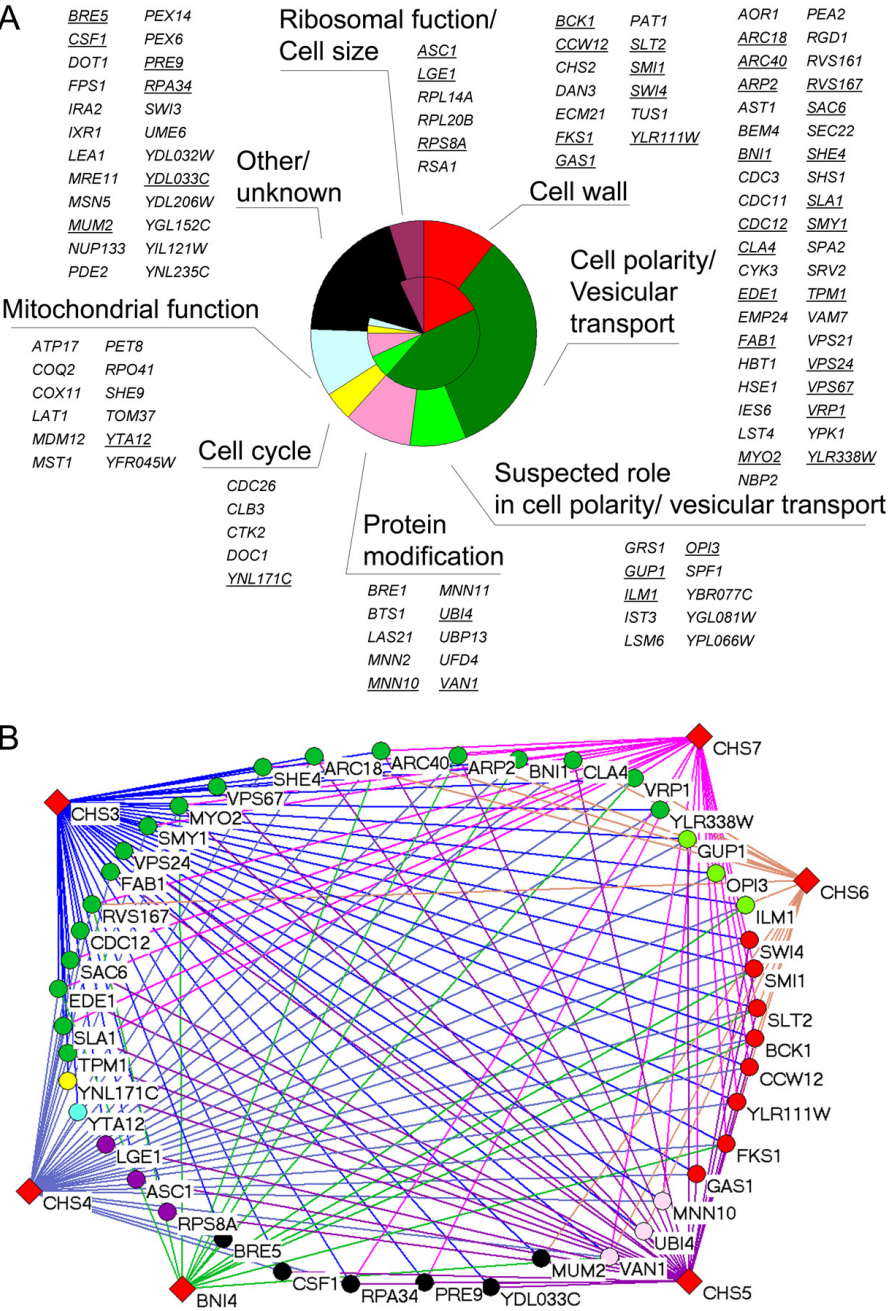
**Analysis of the CSIII network. **(A) Grouping genes of the CSIII network in functional categories. Genes belonging to the "core" group (*CHS3 *plus at least one other) are underlined. The proportions of the functional categories in the CSIII network and in the "core" group are represented in the outer and the inner pies, respectively. (B) Interactions among the "core" group. Color coding for nodes is as in (A).

The 123 genes engaged in the 259 interactions of the CSIII network were grouped by function (Figure 2A, outer pie). Some genes show multiple connections, with 55 of these accounting for almost 75% of the interactions. Among this group, 44 genes (Figure 2B) interact with *CHS3 *and at least one other query gene, reflecting the central role of *CHS3 *in the network. These 44 genes, involved in 166 interactions, are significantly more connected to the query genes than the remaining 11 multiply connected genes, which have 25 interactions (*p *< 0.01). Thus, this set of 44 genes defines a core group of multiply interacting genes. In addition, the "core" genes account for 57 of 74 synthetic lethal interactions of the CSIII network [19], highlighting their importance for survival when the CSIII pathway is defective. Grouping "core" genes by functional categories (Figure 2A, inner pie) revealed enrichment for certain functions relative to the overall CSIII network. For example, cell wall assembly and secretory pathway polarization/vesicular transport contain 18% and 43% of "core" genes, respectively, whereas these functional categories represent 10% and 33% of genes in the CSIII network, respectively. In contrast, the category "mitochondrial function" is under-represented in the "core" group when compared to the CSIII network (Figure 2A). Thus, analysis of the "core" of highly connected genes indicates that cell wall assembly and polarization of the secretory apparatus are central processes buffering defects in the CSIII pathway.

### *CHS1 *interaction set

Some 57 genes show synthetic interactions with *CHS1 *(Figure 1A and Table 2), and while a number of these are embedded in the CSIII network, most interact only with *CHS1*, indicative of a distinct functional role for Chs1p that is analyzed further in the Discussion.

### Chitin content in mutants of interacting genes

To investigate the relationship between the interacting genes and chitin synthesis, the chitin content of the 156 deletion mutants in non-essential genes of the CSIII network and the *CHS1 *interacting genes was measured (see Additional file 2). To focus on the biologically meaningful changes in chitin level, a set of mutants with marginally altered chitin content were excluded from our analysis despite their having statistical significance. Thus, 51 and 3 mutants with levels above 20 and below 12 nmole GlcNAc/mg dry weight, respectively are discussed below as having altered chitin content. To integrate synthetic interaction and chitin determination data, each node of the interaction network was colored according to the chitin level of its deletion mutant (Figure 1A). Four groups of genes emerged from this analysis.

**Figure 3:**
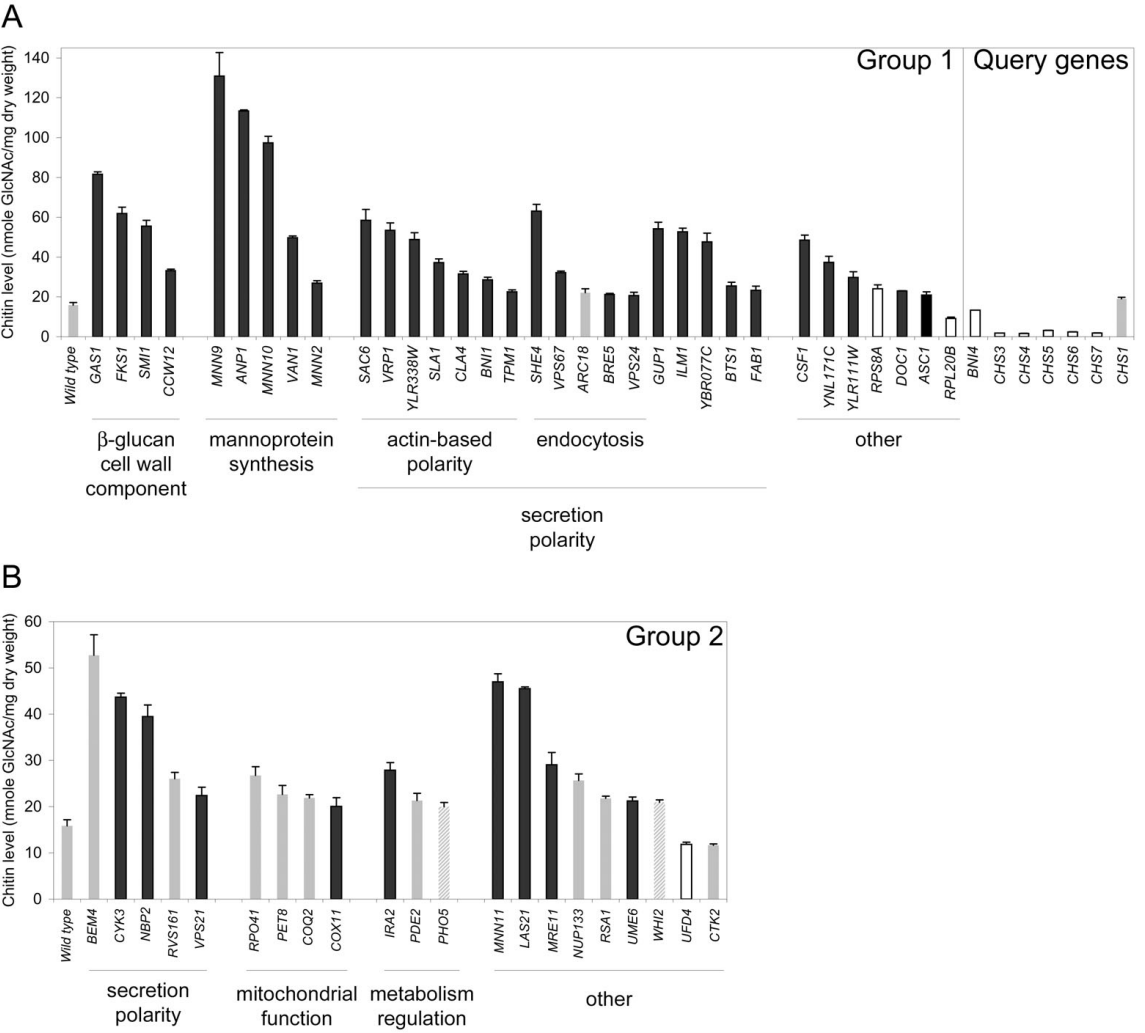
**Grouping deletion mutants with an altered chitin content according to their interaction pattern. **(A) Chitin levels, expressed in nmole GlcNAc/mg dry weight, in wild type, group 1 and query mutants. (B) Chitin levels in wild type and group 2 mutants. Note the different scales in (A) and (B). Hypersensitivity, resistance, wild type and not determined sensitivity to Calcofluor are indicated by black, open, gray and hatched bars, respectively.

Group 1 has 33 mutants with an altered chitin level and a requirement for Chs3p function for optimal growth (Figure 3A). All but one of the group 1 mutants have elevated chitin levels, indicating that they trigger the chitin stress response. Nine of these genes are involved in the synthesis of cell wall components such as β-glucan and mannoprotein. Half of the group 1 genes (17/33) are required for polarization of the actin cytoskeleton or have a function in vesicular transport through retrograde transport in the endosomal pathway. The majority of the group 1 genes belong to the CSIII network "core", with just 7 genes interacting uniquely with *CHS3 *(*ANP1*, *BTS1*, *DOC1*, *MNN2*, *MNN9*, *RPL20B *and *YBR077C*). Thus, the deletion mutants of group 1 genes are highly sensitive to CSIII pathway perturbation.

Group 2 is composed of 19 and 2 mutants with an increased and a decreased chitin level, respectively, but whose optimal growth does not require *CHS3 *(Figure 3B). A large fraction of group 2 genes (16/21, 76%) interact with *CHS5 *and/or *CHS6 *(Table 2). Group 2 mutants are affected in secretion or mitochondrial functions and in the regulation of transcription and translation. The elevated synthesis of chitin in 19 of the group 2 mutants is probably triggered as a non-specific stress response to the mutation, but unlike group 1, it does not serve to buffer against the deleterious effects of the mutation. For example, a set of 14 group 2 mutants interacting with *CHS5 *or *CHS6 *have elevated chitin levels. In these cases, the stress activated chitin response reflects a broader Chs5p- and/or Chs6p-dependent activation that is required for cell wall buffering in these mutants (see Discussion).

**Figure 4:**
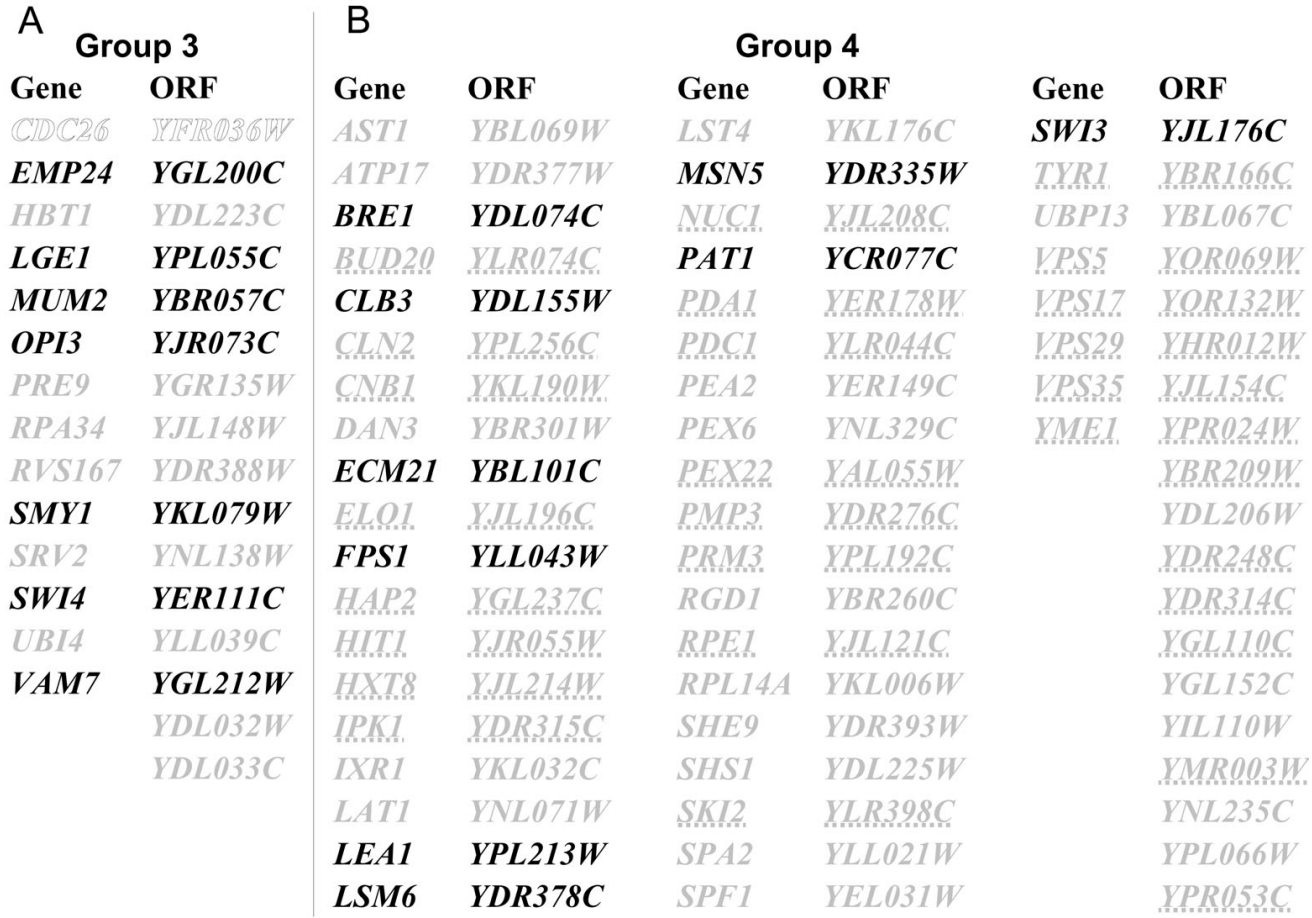
**Grouping deletion mutants with wild type chitin content according to their interaction pattern. **(A) and (B) group 3 and 4 mutants, respectively. Hypersensitivity, resistance, wild type and not determined sensitivity to Calcofluor are indicated by bold, open, gray and underlined characters, respectively.

The third group of 16 mutants have a wild type chitin content and a synthetic interaction with *CHS3 *(Figure 4A). These mutants are defective in ubiquitin processing/cell cycle progression, membrane biogenesis and polarized secretion.

Finally, group 4 contains deletion mutants in 57 genes with a wild type chitin level and a synthetic interaction with any *CHS *gene other than *CHS3 *(Figure 4B). Sixteen of these genes are connected to *CHS5*, suggesting a broad, and Chs3p-independent, role for Chs5p in their buffering.

### Calcofluor white phenotypes of the CSIII network mutants

Calcofluor white is a toxic compound which binds primarily to chitin in yeast, and mutants with cell surface defects frequently show altered sensitivity to it [20-23]. For example, a *chs3 *null mutant and mutants with a defective CSIII pathway show Calcofluor resistance because they make low levels of cell wall chitin [23]. We thus searched for synergistic interactions between Calcofluor white and the deletion of each gene found in the CSIII network. Mutant strains were spotted on solid medium containing 10 μg/ml or 50 μg/ml Calcofluor white, and scored for sensitivity relative to the wild type. In all, 59% of mutants exhibited an altered Calcofluor sensitivity, with 65 and 4 mutants showing hypersensitivity and resistance, respectively (Figure 3 and 4, and see Additional file 2). As seen in Figure 3, a high fraction of mutants with an altered chitin content also showed an altered sensitivity to Calcofluor. Indeed, 80% (39/49) and 67% (2/3) of mutants with increased and decreased chitin levels, respectively, were hypersensitive and resistant to Calcofluor, respectively. More specifically, 97% of group 1 mutants had a Calcofluor phenotype, revealing the critical role of Chs3p-synthesized chitin in Calcofluor sensitivity. However, Calcofluor toxicity does not correlate strictly with the chitin level or the requirement for Chs3p function. Indeed, 10 mutants with wild type Calcofluor sensitivity have an altered chitin content (Figure 3, gray bars). This set is almost entirely composed of group 2 mutants (Figure 3B), with the optimal growth of 9 of these mutants not requiring *CHS3*. In addition, 17 mutants with an altered Calcofluor sensitivity have a wild type chitin level (Figure 4, bold and open characters): 8 and 9 of those mutants fall in groups 3 and 4, respectively. The 8 group 3 mutants require Chs3p function but do not trigger the chitin stress response, indicative of a requirement for an additional Chs3p function distinct from lateral wall chitin synthesis, such as remedial septum or bud neck chitin synthesis (see Discussion). The 9 group 4 mutants require integrity of the CSIII pathway but not an increase of chitin level through Chs3p. This subgroup indicates that components of the CSIII pathway function in other cellular processes. Finally, a set of 26 mutants are wild type for both Calcofluor sensitivity and chitin level. Nineteen of them are not connected to *CHS3*, reflecting chitin-independent functional requirements for *CHS4*, *CHS5*, *CHS6*, *CHS7 *and *BNI4*.

### Synthetic interactions with *SHC1*

**Figure 5:**
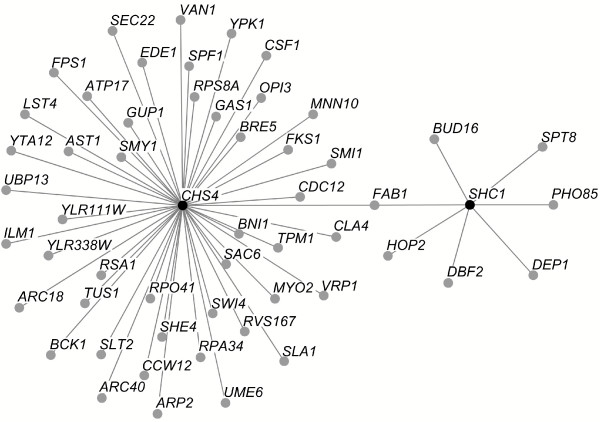
**Comparative synthetic interaction patterns of *CHS4 *and *SHC1*. **Synthetic interactions with *CHS4 *or *SHC1 *are depicted as connections between these nodes and their respective partners (black and gray nodes, respectively).

The *SHC1 *gene product is 43% identical to Chs4p. While Chs4p functions in Chs3p activation during vegetative growth, the known role of Shc1p is restricted to sporulation [24]. However, overexpression of Shc1p during vegetative growth can compensate for the lack of Chs4p, and reciprocally, overexpression of Chs4p during sporulation partially complements the *shc1*Δ mutant phenotype [24]. Although Chs4p and Shc1p show structural and functional relatedness they are not an essential redundant pair since the *chs4 shc1 *double mutant has no synthetic growth defect. We searched for genes required for the optimal vegetative growth of the *shc1*Δ mutant and found 6 synthetic interactions. In addition, we added the previously reported synthetic interaction between *PHO85 *and *SHC1 *[25] to this list. *FAB1 *and *DEP1 *are part of the CSIII network and *CHS1*-interacting set, respectively. The remaining 5 genes (*BUD16*, *DBF2*, *HOP2*, *PHO85 *and *SPT8*) interact uniquely with *SHC1*. The *pho85 *null mutant was not further analyzed due to its very poor growth. The amount of chitin produced in the 4 remaining mutants was measured and found to be similar to the wild type (see Additional file 2). Genes compensating for a *SHC1 *deletion form a distinct group from those buffering a *CHS4 *deletion (Figure 5), and their genetic interactions with *SHC1 *appear to be independent of a chitin defect. Thus, our synthetic interaction data indicate that *SHC1 *has evolved new functions that are not shared with *CHS4 *and which extend the role of Shc1p beyond sporulation to mitotic growth.

## Discussion

We globally analyzed a network of 259 interactions among 123 genes required for optimal growth of *BNI4*, *CHS3*, *CHS4*, *CHS5*, *CHS6 *or *CHS7 *deletion mutants. The query genes are highly interconnected, reflecting common requirements in the *bni4 *and *chs3-7 *null mutants. This network centers on *CHS3 *function, with *CHS3 *sharing most of its interactions with the other query genes.

### Grouping *CHS3*-interacting genes by functional requirement for Chs3p

The genetic interactions observed with *CHS3 *can be sorted by Chs3p function, which includes synthesis of chitin in the lateral wall, in the remedial septum and at the bud neck.

#### Lateral wall chitin, the chitin stress response

**Figure 6:**
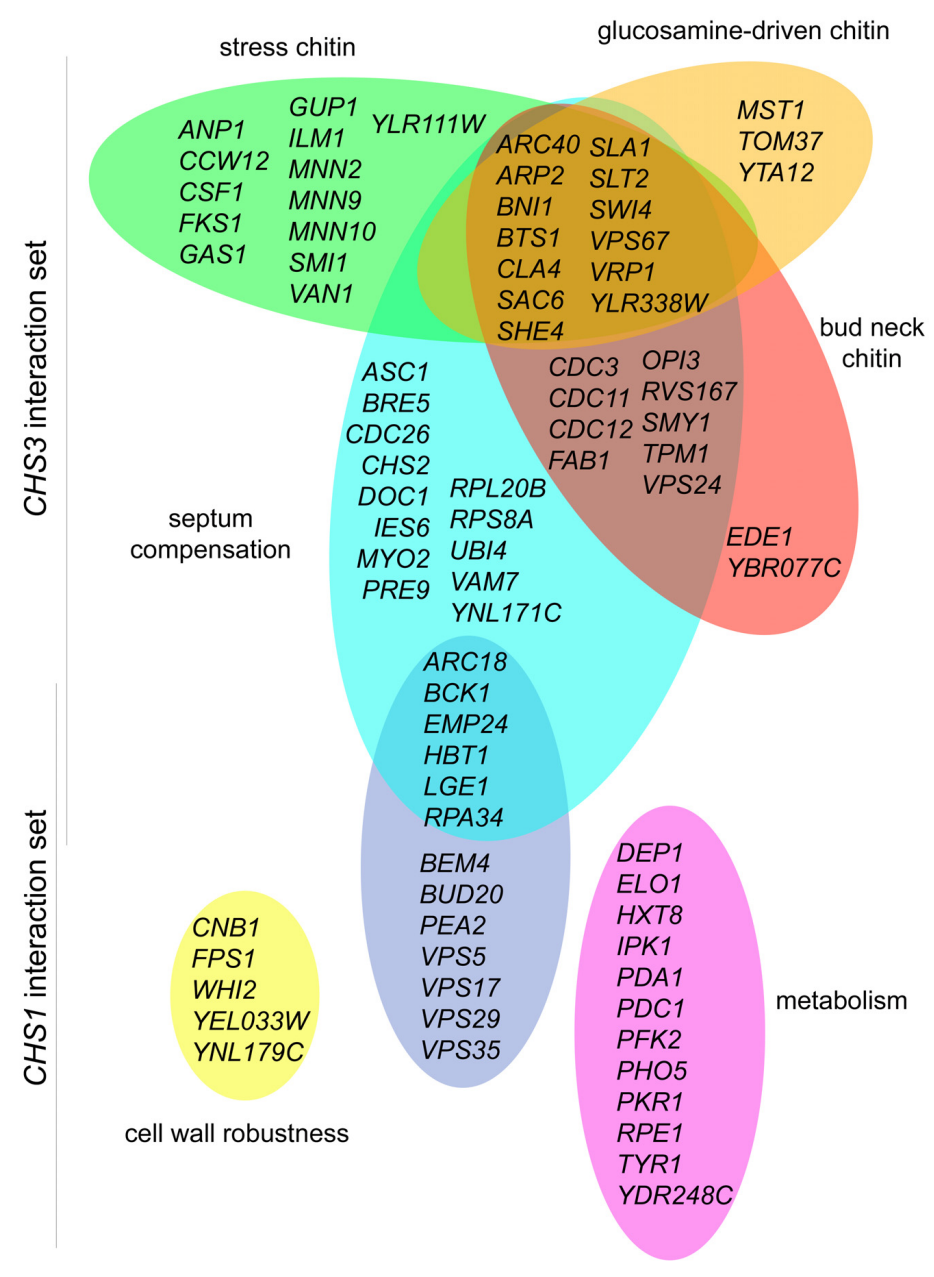
**Functional integration of *CHS1*- and *CHS3*-interaction sets. ***CHS1*- and *CHS3*-interacting genes were grouped according to their effects on chitin synthesis. The Venn diagram shows the distinct and overlapping sets for each functional category.

The Chs3p-dependent synthesis of wall chitin is dramatically stimulated upon cell wall stress, through a stress response pathway involving activation of the chitosome and stimulation of the cell integrity pathway [10,15-17]. Mutants with cell wall defects activate this stress pathway and our synthetic analysis indicates that many of them require Chs3p function (Figure 6). Our work indicates that the extent of this stress response is far greater than previously realized: just 6 of the 26 mutants in this group were previously known to have an altered chitin content. Among such new mutants involved in triggering the chitin stress response are the cell wall protein encoding gene *CCW12*, and the actin-based polarity genes *BNI1*, *CLA4*, *SAC6*, *SHE4*, *SLA1 *and *VRP1*. Actin patches are crucial for the proper targeting of cell wall synthesis components [26], and their perturbation activates a chitin stress response. Other mutants include *CSF1*, *GUP1 *and *ILM1 *that have growth defects on non-fermentable carbon sources and the putative vacuolar protein encoding *YBR077C*.

#### Glucosamine-driven chitin synthesis

Chs3p has an additional role in the synthesis of chitin upon glucosamine addition [27]. The basis for this process is uncertain, but probably relies on metabolic flux changes and appears to be independent of the classic chitin stress response [27]. Deletion of genes compensating for defects in this glucosamine-response pathway may interact synthetically with *CHS3 *and genes of the CSIII pathway (Figure 6). Candidate genes are *MST1*, *TOM37 *and *YTA12*, involved in mitochondrial function, a process known to be down-regulated by glucosamine exposure: their deletion may lead to metabolic imbalance compensated for by an increased chitin synthesis.

#### Insight into Chs2p function

Chs2p is responsible for synthesis of the primary septum but a detailed understanding of how this is achieved remains incomplete. Analysis of *CHS3 *synthetic interactions can give insight into Chs2p function as, in its absence, Chs3p can partially compensate by forming a "remedial septum" [28]. We reasoned that a set of synthetic interactions with *CHS3 *could occur through perturbation of *CHS2 *function, leading to the need for *CHS3*. A group of genes affecting cell cycle progression likely have an impact on septation in this way. For example, mutants in *CDC26*, *DOC1 *or *YNL171C *(which is an *apc1 *allele) show a delay in exit from mitosis and mutants in *ASC1*, *IES6, LGE1, RPL20B, RPS8A *or *VAM7 *exhibit altered cell size, a phenotype frequently reflecting defects in cell-cycle checkpoints [29,30]. Deletion of any of these genes can uncouple cell-cycle progression and septation, resulting in defective synthesis of the primary septum by Chs2p. The synthetic interactions between these genes and *CHS3 *likely result from a failure to fully synthesize both the primary septum (as a consequence of a defect in cell-cycle progression) and a remedial septum (Figure 6). Pertinently and consistent with our data, Ufano *et al*. [31] show that deletion of *SWM1*, encoding a subunit of the anaphase promoting complex, also leads to an increase of Chs3p-catalyzed chitin deposition.

Chs2p has a cryptic *in vitro *activity that can be detected only after treatment of a cell extract with trypsin. This suggests that Chs2p may also be produced as a zymogen *in vivo *and be activated by posttranslational modification [32]. Although proteomic analysis reveals the existence of ubiquitinated and phosphorylated forms of Chs2p [33,34], the effect of these modifications on Chs2p activity is unknown. Mutants with defects in Chs2p activation or turnover may exhibit a low Chs2p activity and depend on a compensatory Chs3p activity. Of the *CHS3 *interacting genes, the serine/threonine protein kinases Bck1p and Slt2p are candidates for Chs2p activation, while the polyubiquitin gene *UBI4*, the ubiquitin protease Bre5p and the proteasome subunit Pre9p may be required for Chs2p turnover (Figure 6).

#### Chitin at the bud neck

Chs3p synthesizes a chitin ring that marks the incipient bud site. Defects in secretion or polarization of the secretory apparatus may lead to abnormal bud neck assembly and/or septation. For example, the genes *EDE1*, *EMP24*, *FAB1*, *HBT1*, *OPI3*, *RVS167*, *SMY1*, *TPM1*, *VPS24*, *VPS67 *or *YBR077C *are required for polarization of the secretory pathway, indicating that transport and proper localization of protein(s) to the bud neck are essential for growth of mutants with low CSIII activity. Thus, we identify these genes as candidates for involvement in Chs2p localization and in bud neck integrity (Figure 6).

### Functions of Bni4p, Chs4p and Chs5p beyond chitin synthesis

The existence of synthetic interactions with *BNI4*, *CHS4*, *CHS5 *or *CHS6 *not shared with *CHS3 *uncovers functions of these genes that are unrelated to Chs3p transport or activity.

#### Bni4p

Five genes interact uniquely with *BNI4*, indicating that Bni4p has functions distinct from anchoring Chs3p to the septin ring. Among these, *NBP2*, *RGD1*, *SHS1 *and *SPA2 *are required for regulation of cytoskeleton organization at the bud neck by the cell integrity pathway. Further, *BNI4 *shows a unique interaction with *YPL066W*, which together with the bud neck localization of Ypl066p [35] implicates this gene in bud development. Our data and the finding that localization of Crh2p at the bud neck requires Bni4p [36] indicate that Bni4p has a broad role in bud neck organization.

#### Chs4p

Of the 7 unique *CHS4 *interacting genes, 4 are required for trafficking of membrane proteins. Ast1p and Lst4p are required for Golgi to plasma membrane transport of the H^+^-ATPase Pma1p and the amino-acid permease Gap1p, respectively [37,38]. Spf1p, a putative calcium pump of the endoplasmic reticulum, may also play a role in the translocation of transmembrane proteins [39]. Ypk1p, a serine/threonine protein kinase required for full induction of the *PKC1-SLT2 *cell integrity pathway under stress condition, is also required for endocytosis [40]. Absence of these genes combined with a *CHS4 *deletion likely leads to defects in targeting membrane proteins to the septin ring, with resultant synthetic growth phenotypes.

#### Chs5p and Chs6p

Chs5p and Chs6p are late-Golgi localized proteins involved in targeting Chs3p to sites of polarized growth [12] and to the plasma membrane [13]. Our results for *CHS5 *and *CHS6*, showing a strong web of synthetic interactions with CSIII network "core" genes, reflect these roles. Whereas little is known about a Chs6p function besides Chs3p trafficking, Chs5p is also involved in the selective polarization of other surface proteins, such as Fus1p [41] and, at least partially, Crh2p [36]. *CHS5 *and *CHS6 *show a large number of *CHS3*-independent interactions (39/71 and 9/25, respectively), suggesting multiple additional roles for Chs5p and Chs6p in protein targeting. Interestingly, a number of these interacting mutants have elevated chitin levels and fall into group 2. For example, the mutants *ira2 *and *pde2 *are synthetic with *CHS5 *and *CHS6 *and *CHS5*, respectively and make 76% and 35% more chitin than the wild type, respectively (Figure 3B). These mutants constitutively elevate the Ras/cAMP pathway and *pde2 *mutants are known to affect cell wall integrity and to cause slight changes in glucan and chitin levels [42,43]. Our work suggests that the chitin elevation involves increased activity of the Chs5p and Chs6p chitosome pathway. However, the key buffering component in this cAMP response is not chitin, but must be some other component of the activated chitosome pathway, since neither *ira2 *nor *pde2 *show a synthetic interaction with *CHS3*. Thus here, the stress activated chitin response is a gratuitous consequence of a broader Chs5p- and/or Chs6p-dependent activation that is required for cell wall buffering in these mutants.

Regarding the known role of Chs5p in specialized late-Golgi trafficking; several of the *CHS5*-interacting genes have products that likely work in conjunction or in parallel with Chs5p. These include *AOR1*, *BEM4*, *HSE1*, *LSM6*, *PEA2*, *RVS161*, *SEC22 *and *VPS21*. A new candidate is *YGL081W *that interacts with *CHS5*, and whose product has been found in a complex containing Cop1p, required for Golgi retrograde transport [44].

*CHS5 *interacts uniquely with 6 genes involved in mitochondrial function (*COQ2*, *COX11*, *LAT1*, *MDM12*, *PET8 *and *SHE9*) some of which show elevated chitin levels. These mitochondrial proteins may play indirect roles in late-Golgi trafficking; for example, the secretory apparatus and mitochondria exchange lipids [45], and a defect in mitochondrial function may impact on secretory function. Alternatively, in the absence of *CHS5*, mitochondria may be poorly transferred to daughter cell with their efficient functioning being essential for optimal growth.

Finally, some 9 genes show an interaction with both *CHS5 *and *CHS1 *(Figure 1A), indicating some common requirement for these genes. One provocative possibility for this interactional signature is that Chs5p is involved in the targeting of Chs1p.

### Analysis of synthetic interactions with *CHS1*

Although it was the first fungal chitin gene identified, the role of Chs1p has remained unclear. Cell lysis phenotypes of *chs1 *mutants have led to the view that Chs1p is an "auxiliary" enzyme implicated in the repair of chitinase-mediated cell wall damage associated with cell separation [7]. How such damage is sensed or how the repair process is activated remains unclear. The line between repair and redundant synthesis with Chs2p may be an arbitrary one, and a direct role for Chs1p involvement in septal chitin synthesis on growth in acidic minimal media where cell lysis is more pronounced, also explains the phenotype (see [46] for a discussion). The lysis phenotypes associated with *CHS1 *deletion also show strain variability. For example, a strain with a recessive suppressor in an uncharacterized gene *SCS1 *shows no lysis phenotype, indicating the involvement of other genes [7].

Our synthetic approach allows a broad survey of possible *CHS1 *function. However, *CHS1 *is part of a family and a synthetic analysis of a gene family can be complicated [47]. Specialized roles for *CHS1*, *CHS2 *and *CHS3 *are likely ancient, predating the genome duplication of *S. cerevisiae *[48,49], since all three genes are present in *Ashbya gossypii*, a related fungus that did not undergo the *S. cerevisiae *duplication event. Our finding that the majority of the *CHS1 *interactions are both distinct from the CSIII network and do not trigger the chitin stress response (Figure 1A) indicates distinct function. *CHS1 *and *CHS3 *mutants do not synthetically interact under our test conditions, so the synthetic effects of *CHS1 *mutants are not caused by a buffering of *CHS3 *function. Consistent with this, the *CHS1 *deletion does not activate the chitin stress response, as chitin levels in the *chs1*Δ mutant are close to wild type [6], see Figure 3A). One possible cause of synthetic effects of *CHS1 *mutants is through genes that buffer Chs2p function. A number of unique interactors with *CHS1 *are involved in bud morphogenesis (*BEM4*, *BUD20*, *PEA2*), and in protein recycling through the endocytic pathway (*VPS5*, *VPS17*, *VPS29 *and *VPS35*), all could be required for Chs2p function (Figure 6). This hypothesis, supported by the genetic evidence presented here, will require further testing.

In our data we also find interactions with mutants in a number of genes that are singly prone to lysis or show phenotypes consistent with osmotic imbalance (*ARC18*, *BCK1*, *CNB1*, *FPS1*, and *WHI2*). *CHS1 *also shows synthetic interactions with *YEL033W *and *YNL179C*, that overlap with and are alleles of *YEL034W/HYP2 *and *YNL180C/RHO5*, genes that play a role in balancing cell integrity [50,51], and with *YOR322C *which has a role in signaling through the cell integrity pathway [52]. In addition the absence of Chs1p is buffered by the presence of 16 genes (*BCK1*, *BEM4*, *CNB1*, *ECM21*, *FPS1*, *GRS1*, *HBT1*, *HIT1*, *NUC1*, *PDA1*, *PFK2*, *SPF1*, *TYR1*, *YGL081C*, *YGL110C *and *YPL261C*) that show synthetic interactions with the *FKS1*, *GAS1 *or *SMI1 *genes involved in β-1, 3-glucan synthesis [19]. These results provide strong independent support for a function of Chs1p in buffering cell wall robustness through regulated chitin synthesis, and identify many candidates that may participate in the modulation of Chs2p function.

As mentioned above, yeast cells are more dependent on Chs1p to prevent lysis and allow growth on synthetic minimal media [6,53]. The basis for this increased dependence is unknown, though there are data indicating that the partitioning of Chs1p activity between the plasma membrane and the chitosome is somewhat more pronounced toward the plasma membrane in minimal medium [54]. Interestingly, a number of unique *CHS1 *interactors are involved in metabolism and nutrient utilization (Figure 6), providing functional clues to this aspect of Chs1p function.

## Conclusions

Our synthetic network analysis reveals a deep interactional complexity underlying chitin biology. The *CHS3*-core network is informative in identifying components involved in all aspects of regulated chitin deposition. The chitin stress response that adds chitin to lateral cell walls is now shown to be triggered very broadly by cell wall and actin-based polarity defects and to play a key role in cell wall buffering. The *CHS3 *core-network also offers insight into Chs2p function by identifying proteins implicated in bud neck localization, and in the cell cycle coordination of septum formation with mitotic exit. Genes involved in secretory trafficking of Chs3p (*CHS4*, *CHS5*, *CHS6 *and *BNI4*) show many *CHS3*-independent interactions and these greatly expand the range of trafficking functions for these genes, especially for the heavily interacting *CHS5*. In contrast to its currently assigned minor auxiliary role, *CHS1 *shows an extensive web of genetic interactions, most of which are distinct from the CSIII network and which do not trigger the chitin stress response. One set of these identifies components of endocytosis, budding and cell morphology, which may be required for Chs2p function. A second set of 25 interacting genes show that Chs1p is intimately involved in buffering yeast cell wall robustness during vegetative growth.

## Methods

### Strains, media and drugs

Haploid deletion mutants (Table 1) are available from the deletion project consortium. These strains were arrayed on sixteen 768-format plates using a colony picker [18]. Starting strains for the SGA analysis (Table 1) were constructed as described in Tong *et al. *[19]. Arrays were propagated at 30°C on standard YEPD (10 g/l yeast extract, 20 g/l bacto-peptone, 20 g/l glucose) or YEPD supplemented with 200 μg/ml G-418 (Invitrogen, Carlsbad, CA). When required, strains were grown on standard SD medium (6.7 g/l yeast nitrogen base, 20 g/l glucose) supplemented with appropriate amino acids [55]. Nourseothricin (ClonNat) was purchased from Werner Bioagent (Jena, Germany).

**Table 1:** Strains used in this study.

**Strain**	**Genotype**	**Reference**
BY4741	*MATa his3*Δ *leu2*Δ *met15*Δ* ura3*Δ	[60]
BY4742	*MATα his3*Δ *leu2*Δ *lys2*Δ* ura3*Δ	[60]
BY4743	*MATa/α his3*Δ */his3*Δ *leu2*Δ */leu2*Δ *met15*Δ */MET15 lys2*Δ */LYS2 ura3*Δ */ura3*Δ	[60]
ΔarrayORF	*MATa orf*Δ*::KanMX4 his3*Δ *leu2*Δ *met15*Δ *ura3*Δ	[61]
HAB1122	As Y3656 *chs3*Δ*::NatMX4*	[19]
SBY4	As Y3084 *chs1*Δ*:: NatMX4*	[19]
SBY5	As Y3084 *chs5*Δ*:: NatMX4*	[19]
SBY6	As Y3084 *chs7*Δ*:: NatMX4*	[19]
SBY30	As Y3084 *chs4*Δ*:: NatMX4*	[19]
SBY70	As Y3084 *shc1*Δ*::NatMX4*	This work
SBY105	As Y3656 *chs6*Δ*:: NatMX4*	[19]
Y3084	*MATα mfα1*Δ *::MFα 1pr-LEU2 can1*Δ *::MFA1pr-HIS3 his3*Δ *leu2*Δ *lys2*Δ *ura3*Δ	[18]
Y3638	As Y3084 *bni4*Δ*:: NatMX4*	[19]
Y3656	*MATα can1*Δ *::MFA1pr-HIS3-MFα 1pr-LEU2 his3*Δ *leu2*Δ *lys2*Δ *ura3*Δ	[19]

### Screening for synthetic lethal/sick interactions and data refinement

Synthetic genetic array analysis (SGA) was used to identify genes required for the optimal growth of strains deleted for *BNI4*, *CHS1*, *CHS3-7 *or *SHC1*, as described [18,19]. From three SGA screens for each "query" gene, ~1,800 potential interactions were identified and 333 synthetic interactions confirmed by random spore or tetrad analysis as described previously [19]. Briefly, spores were germinated into liquid haploid selection medium [SD-His/Arg + canavanine] in a 96-well format. The germinated *MATa *spore progeny were serially diluted in sterile water and 2 μl for each dilution was spotted onto medium selecting for the query-gene mutation [SD-His/Arg + canavanine/Nourseothricin], the interacting gene mutation [SD-His/Arg + canavanine/G-418], and both the query-gene and interacting gene mutations [SD-His/Arg + canavanine/Nourseothricin/G-418] then incubated at 30°C for ~2 days. Cell growth under the three conditions was compared and double mutants were scored as synthetic sick (SS), synthetic lethal (SL) or no interaction (No) [19]. For tetrad analysis, dissections were performed on solid complete SD medium and growth of individual spores was scored after 4 days incubation at 30°C. Plates were then replicated on YEPD + G-418 or Nourseothricin to identify tetrad type. Growth of double mutants was compared to that of single mutants from tetratype tetrads and then scored as "SS", "SL "or "No". Ten of the 22 previously reported synthetic lethal interactions with *CHS3-7 *or *SHC1 *[4,15,25,56,57] were found by the SGA procedure. Of the remaining, 9 engaged genes whose mutant is absent from our deletion collection (*CDC3*,*CDC11*, *CDC12 *and *CHS2*) or genes whose deletion leads to systematic growth defects in our conditions (*ANP1*, *MNN9*, *PHO85 *and *SRV2*) and these genes were used in our network analysis. No synthetic interaction between *GAS1 *and *CHS4 *or *CHS7 *was found by the SGA. These discrepancies with other's data [56] reflect differences in strain background. These two synthetic interactions were included in our analysis. An additional set of 57 interactions were analyzed further by random spore or tetrad analysis [19]. Of these, 30 synthetic interactions were confirmed, with the 27 remainder discarded (see Additional file 1). It is important to note that this additional set of tested interactions was not random and was strongly biased toward dubious interactions: for example, a group of 11 interactions with genes closely linked to *CHS1 *or a set of 16 non-reciprocal interactions (that is gene A found in screen for genes interacting with gene B and gene B not found in the set of genes interacting with gene A).

### Chitin assay

Stationary phase cultures were diluted 1:100 into 3 ml of YEPD and grown again for 22-24 h at 30°C. Cells from 1.5 ml culture were colleted by centrifugation (20,000 × g, 2 min). Pellets were then frozen at -20°C until used for alkali-extraction. Dry weights were determined after a 2 day incubation at 37°C. Cell pellets were resuspended in 1 ml 6% KOH and heated at 80°C for 90 min with occasional mixing. Alkaline insoluble material was pelletted (20,000 × g, 20 min), neutralized with phosphate-buffered saline for 10–20 min with occasional mixing. After centrifugation (20,000 × g, 20 min), 200 μl of McIlvaine's Buffer (0.2 M Na_2_HPO_4_/0.1 M citric acid, pH 6.0) was added to pellets. Extracts were then stored at -20°C until processed for chitin measurements. Samples were thawed and subjected to two digestions with 4 μl of purified *Streptomyces plicatus *chitinase-63 (4 μg/ul in PBS) at 37°C for 36–40 h and then for 20–24 h. The amounts of chitin were then determined by using the modified Morgan-Elson procedure as described previously [27]. The levels of chitin, expressed as GlcNAc concentration, were then normalized to the dry weight of the sample. Of the 84 mutants whose chitin levels differed significantly from wild type (*p *< 0.01 in a Student's t-test, see Additional file 2), 54 with larger changes were further considered (see text).

### Calcofluor white sensitivity/resistance

Sensitivity to Calcofluor white was assessed using a modified version of the method described by Ram *et al. *[21]. Cells were grown overnight, and then diluted to an optical density of OD_600 nm _= 0.5. Five μl of this suspension, as well as 1:10, 1:100, and 1:1000 dilutions of this suspension, were spotted on SD plates (buffered to pH 6.2 with 10 mM MES) containing 10 μg/ml or 50 μg/ml Calcofluor white (Fluorescent Brightener 28, Sigma), and control plates. Plates were incubated at 30°C for 48 hours, photographed, and then rechecked after 72 hours. A literature search indicated that the phenotype we found agreed with that previously reported for 29 mutants (22 interacting mutants + 7 query mutants). In 4 cases however (*ARC18*, *PDE2*, *PEX6 *and *SPF1*), we found a wild type sensitivity for mutants that had previously shown altered Calcofluor white sensitivity [22,42,58,59]. These discrepancies may be due to differences in Calcofluor white concentration or to allelic or strain variation.

## Authors' contributions

GL participated in the design of the study and its coordination, collected and analyzed data, and drafted the manuscript. JS carried out confirmations of SGA screens and Calcofluor white sensitivity assays. CAS carried out chitin determinations and data analysis. AMS, PM, SH and AHYT carried out SGA screens. HB and CB conceived the study, and oversaw its design and coordination. HB participated in data analysis and manuscript writing. All authors read and approved the final manuscript.

## Supplementary Material

Additional File 1 Title: Interactions reported previously and not included in our global analysis. Click here for file

Additional File 2 Title: Sensitivity to Calcofluor white and chitin levels of mutants. Click here for file
