# Supplementary material for: An interactional network of genes involved in chitin synthesis in Saccharomyces cerevisiae
Source: BMC Genet. 2005 Feb 16;6:8. doi: 10.1186/1471-2156-6-8 (PMC554099; doi:10.1186/1471-2156-6-8)
Supplement: Additional File 1 — Title: Interactions reported previously and not included in our global analysis. [file 1471-2156-6-8-S1.pdf]

Supplementary Table 1. Interactions reported previously (Tong *et al.*, 2004, *Science* **303:**808-813) and not included in our global analysis.

**Query**	**gene**	**ORF**	**Confirmation procedure**	**Explanation**
BNI4	*SMY1*	*YKL079W*	RSA[(1)]	non reciprocal interaction[(2)]
CHS1		*YNL087W*	tetrad analysis	linked to *CHS1*
CHS1		*YNL171C*	tetrad analysis	linked to *CHS1*
CHS1		*YNL200C*	tetrad analysis	linked to *CHS1*
CHS1		*YNL201C*	tetrad analysis	linked to *CHS1*
CHS1		*YNL203C*	tetrad analysis	linked to *CHS1*
CHS1		*YNL228W*	tetrad analysis	linked to *CHS1*
CHS1		*YNL235C*	tetrad analysis	linked to *CHS1*
CHS1	*FIG2*	*YCR089W*	tetrad analysis	non reciprocal interaction
CHS1	*PDR16*	*YNL231C*	tetrad analysis	linked to *CHS1*
CHS1	*PEX6*	*YNL329C*	tetrad analysis	linked to *CHS1*
CHS1	*SPS18*	*YNL204C*	tetrad analysis	linked to *CHS1*
CHS1	*SPS19*	*YNL202W*	tetrad analysis	linked to *CHS1*
CHS3	*KRE1*	*YNL322C*	tetrad analysis	non reciprocal interaction
CHS3	*RVS161*	*YCR009C*	tetrad analysis	non reciprocal interaction
CHS4	*KRE1*	*YNL322C*	tetrad analysis	non reciprocal interaction
CHS4	*RVS161*	*YCR009C*	tetrad analysis	non reciprocal interaction
CHS5	*BBC1*	*YJL020C*	tetrad analysis	non reciprocal interaction
CHS5	*BNI1*	*YNL271C*	tetrad analysis	non reciprocal interaction
CHS5	*EMP24*	*YGL200C*	tetrad analysis	non reciprocal interaction
CHS5	*KNH1*	*YDL049C*	tetrad analysis	non reciprocal interaction
CHS5	*KRE1*	*YNL322C*	tetrad analysis	non reciprocal interaction
CHS5	*RVS167*	*YDR388W*	tetrad analysis	non reciprocal interaction
CHS5	*SMY1*	*YKL079W*	tetrad analysis	non reciprocal interaction
CHS6	*RVS161*	*YCR009C*	tetrad analysis	non reciprocal interaction
CHS7	*RVS161*	*YCR009C*	tetrad analysis	non reciprocal interaction
SHC1	*BIK1*	*YCL029C*	tetrad analysis	non reciprocal interaction

[(1)]: random spore analysis.

[(2)]: a non-reciprocal interaction between genes A and B occurs when gene A is found in a screen for genes interacting with gene B, whereas gene B is not found in the set of genes interacting with gene A.
