# Supplementary material for: An interactional network of genes involved in chitin synthesis in Saccharomyces cerevisiae
Source: BMC Genet. 2005 Feb 16;6:8. doi: 10.1186/1471-2156-6-8 (PMC554099; doi:10.1186/1471-2156-6-8)
Supplement: Additional File 2 — Title: Sensitivity to Calcofluor white and chitin levels of mutants. [file 1471-2156-6-8-S2.pdf]

Supplementary Table2. Sensitivity to Calcofluor white and chitin levels of mutants.

Gene	ORF	CFW phenotype[(1)]	Chitin level[(2)] (nmole GlcNAc/mg dry weight)
wild type[(3)]			15.8 ± 1.4
ANP1	*YEL036C*	s[(4)]	113.3 ± 0.6
AOR1	*YBR231C*	s	18.2 ± 0.3
ARC18	*YLR370C*	wt	22.1 ± 2
ASC1	*YMR116C*	s	20.9 ± 1.6
AST1	*YBL069W*	wt	16.6 ± 0.5
ATP17	*YDR377W*	wt	17.5 ± 1.4
BCK1	*YJL095W*	s	12.5 ± 0.5
BEM4	*YPL161C*	wt	52.7 ± 4.5
BNI1	*YNL271C*	s	28.6 ± 1.3
BNI4	*YNL233W*	r	13.3 ± 0
BRE1	*YDL074C*	s	16.6 ± 0.8
BRE5	*YNR051C*	s	21.3 ± 0.5
BTS1	*YPL069C*	s	25.5 ± 1.9
BUD16	*YEL029C*		17.7 ± 1.2
BUD20	*YLR074C*		17.9 ± 0.2
CCW12	*YLR110C*	s	33.2 ± 0.7
CDC26	*YFR036W*	r	14.6 ± 0.5
CHS1	*YNL192W*	wt	19 ± 0.7
CHS3	*YBR023C*	r	1.8 ± 0.1
CHS4	*YBL061C*	r	1.6 ± 0.2
CHS5	*YLR330W*	r	3.2 ± 0.1
CHS6	*YJL099W*	r	2.4 ± 0.1
CHS7	*YHR142W*	r	1.8 ± 0.1
CLA4	*YNL298W*	s	31.5 ± 1.3
CLB3	*YDL155W*	s	15.3 ± 0.8
CLN2	*YPL256C*		16.6 ± 0.9
CNB1	*YKL190W*		15.4 ± 0.5
COQ2	*YNR041C*	wt	21.9 ± 0.7
COX11	*YPL132W*	s	20 ± 1.9
CSF1	*YLR087C*	s	48.5 ± 2.6
CTK2	*YJL006C*	wt	11.7 ± 0.3
CYK3	*YDL117W*	s	43.7 ± 0.9
DAN3	*YBR301W*	wt	17.3 ± 0.2
DBF2	*YGR092W*		17.6 ± 2.5
DEP1	*YAL013W*		12.6 ± 0.5
DOC1	*YGL240W*	s	22.9 ± 0.2
DOT1	*YDR440W*	wt	13.2 ± 0.8
ECM21	*YBL101C*	s	16.2 ± 0.4

EDE1	*YBL047C*	s	19.5 ± 0.9
ELO1	*YJL196C*		14.6 ± 1.2
EMP24	*YGL200C*	s	14.8 ± 0.9
FAB1	*YFR019W*	s	23.4 ± 2.1
FKS1	*YLR342W*	s	61.9 ± 3.2
FPS1	*YLL043W*	s	17.6 ± 1.4
GAS1	*YMR307W*	s	81.5 ± 1.2
GRS1	*YBR121C*	wt	18.9 ± 0.4
GUP1	*YGL084C*	s	54.2 ± 3.4
HAP2	*YGL237C*		17.8 ± 1
HBT1	*YDL223C*	wt	14.2 ± 1.4
HOP2	*YGL033W*		11.7 ± 0.5
HSE1	*YHL002W*	s	12.6 ± 1.2
HXT8	*YJL214W*		13.8 ± 0.6
IES6	*YEL044W*	s	18.7 ± 1.4
ILM1	*YJR118C*	s	52.7 ± 1.8
IPK1	*YDR315C*		18 ± 0.6
IRA2	*YOL081W*	s	27.9 ± 1.7
IST3	*YIR005W*	wt	13.2 ± 0.6
IXR1	*YKL032C*	wt	15.1 ± 1.3
LAS21	*YJL062W*	s	45.5 ± 0.4
LAT1	*YNL071W*	wt	15.9 ± 0.5
LEA1	*YPL213W*	s	17.3 ± 0.4
LGE1	*YPL055C*	s	17.6 ± 0.5
LSM6	*YDR378C*	s	16.5 ± 1.3
LST4	*YKL176C*	wt	15.8 ± 0.6
MDM12	*YOL009C*	wt	19.3 ± 13.4
MDM38	*YOL027C*		18.4 ± 0.4
MNN2	*YBR015C*	s	26.9 ± 1.2
MNN9	*YPL050C*	s(4)	130.9 ± 11.8
MNN10	*YDR245W*	s	97.3 ± 3.3
MNN11	*YJL183W*	s	47 ± 1.8
MRE11	*YMR224C*	s	29.1 ± 2.6
MSN5	*YDR335W*	s	15.1 ± 0.8
MST1	*YKL194C*	s	19.6 ± 0.9
MUM2	*YBR057C*	s	15.5 ± 0.8
NBP2	*YDR162C*	s	39.5 ± 2.5
NUC1	*YJL208C*		13.7 ± 0.8
NUP133	*YKR082W*	wt	25.7 ± 1.4
OPI3	*YJR073C*	s	17.4 ± 2.3
PAT1	*YCR077C*	s	13.9 ± 0.1
PDA1	*YER178W*		15.1 ± 0.7
PDC1	*YLR044C*		14.1 ± 0.7
PDE2	*YOR360C*	wt	21.3 ± 1.6
PEA2	*YER149C*	wt	13.7 ± 0.9

PET8	*YNL003C*	wt	22.7 ± 1.9
PEX6	*YNL329C*	wt	13.9 ± 0.4
PEX14	*YGL153W*	wt	13.5 ± 0.4
PEX22	*YAL055W*		15.7 ± 0.5
PFK2	*YMR205C*		13.5 ± 1.1
PHO5	*YBR093C*		20 ± 0.9
PKR1	*YMR123W*		18.2 ± 1.8
PMP3	*YDR276C*		14 ± 1.2
PRE9	*YGR135W*	wt	16 ± 1.5
PRM3	*YPL192C*		15.2 ± 0.8
RGD1	*YBR260C*	wt	16.6 ± 1.5
RPA34	*YJL148W*	wt	15.1 ± 0.4
RPE1	*YJL121C*		15.5 ± 0.8
RPL14A	*YKL006W*	wt	14 ± 1.5
RPL20B	*YOR312C*	r	9.2 ± 0.7
RPO41	*YFL036W*	wt	26.7 ± 1.9
RPS8A	*YBL072C*	r	24.1 ± 2
RSA1	*YPL193W*	wt	21.8 ± 0.5
RVS161	*YCR009C*	wt	26 ± 1.4
RVS167	*YDR388W*	wt	17.8 ± 1.4
SAC6	*YDR129C*	s	58.5 ± 5.4
SEC22	*YLR268W*	s	19.2 ± 0.5
SHC1	*YER096W*		14.4 ± 1.7
SHE4	*YOR035C*	s	63.1 ± 3.5
SHE9	*YDR393W*	wt	17.9 ± 0.2
SHS1	*YDL225W*	wt	18.1 ± 1.5
SKI2	*YLR398C*		14.9 ± 1.2
SLA1	*YBL007C*	s	37.2 ± 1.9
SLT2	*YHR030C*	s	12.8 ± 1.1
SMI1	*YGR229C*	s	55.6 ± 2.9
SMY1	*YKL079W*	s	15.1 ± 0.9
SPA2	*YLL021W*	wt	17.1 ± 1.1
SPF1	*YEL031W*	wt	17.9 ± 1.4
SPT8	*YLR055C*		18 ± 0.8
SRV2	*YNL138W*		13.0 ± 0.8
SWI3	*YJL176C*	s	15.2 ± 1.5
SWI4	*YER111C*	s	17.4 ± 1.6
TOM37	*YMR060C*	s	18.8 ± 1.6
TPM1	*YNL079C*	s	22.6 ± 0.9
TUS1	*YLR425W*	s	19.5 ± 1.1
TYR1	*YBR166C*		17.5 ± 0.9
UBI4	*YLL039C*	wt	15.8 ± 1.5
UBP13	*YBL067C*	wt	15.9 ± 1
UFD4	*YKL010C*	r	11.8 ± 0.5
UME6	*YDR207C*	s	21.2 ± 0.9

UTH1	*YKR042W*		12.4 ± 0.9
VAM7	*YGL212W*	s	13.9 ± 0.7
VAN1	*YML115C*	s	49.7 ± 1
VPS5	*YOR069W*		17.6 ± 1.7
VPS17	*YOR132W*		15.8 ± 1.2
VPS21	*YOR089C*	s	22.4 ± 1.8
VPS24	*YKL041W*	s	20.7 ± 1.6
VPS29	*YHR012W*		16 ± 0.7
VPS35	*YJL154C*		16.2 ± 1.6
VPS67	*YKR020W*	s	32.2 ± 0.8
VRP1	*YLR337C*	s	53.5 ± 3.7
WHI2	*YOR043W*		21 ± 0.5
YME1	*YPR024W*		15.7 ± 0.8
YPK1	*YKL126W*	wt	12.7 ± 0.7
YTA12	*YMR089C*	wt	18.5 ± 1.2
	YBR077C	s	47.6 ± 4.5
	YBR209W		14.7 ± 0.2
	YDL032W	wt	18 ± 1.5
	YDL033C	wt	16.9 ± 2.4
	YDL206W	wt	14.1 ± 0.5
	YDR248C		15.5 ± 0.9
	YDR314C		16.9 ± 1
	YEL033W		18.5 ± 1.2
	YFR045W	wt	13.6 ± 0.3
	YGL081W	wt	13.6 ± 0.9
	YGL110C		14.7 ± 0.6
	YGL152C	wt	14.3 ± 0.8
	YIL110W		16 ± 0.3
	YIL121W	wt	12.9 ± 0.5
	YLR111W	s	29.8 ± 2.8
	YLR338W	s	48.9 ± 3.4
	YMR003W		14.1 ± 0.9
	YNL171C	s	37.3 ± 3.1
	YNL179C		13.2 ± 0.9
	YNL235C	wt	17.7 ± 1
	YOR322C		19.3 ± 0.8
	YPL066W	wt	17.4 ± 0.3
	YPL261C		13.1 ± 0.4
	YPR053C		15.9 ± 1.8

(1): Mutants showing increased, decreased or wild type sensitivity to Calcofluor white are scored s, r, or wt, respectively.
(2): Values are an average of at least three independent determinations with a standard deviation. Values statistically higher and lower than wild type ($p < 0.01$) are highlighted in red and green, respectively.

(3): All mutants are isogenic to BY4741 (*MATa his3*Δ *leu2*Δ *met15*Δ *ura3*Δ) except *anp1*Δ and *mnn9*Δ that are isogenic to BY4742 (*MAT*α *his3*Δ *leu2*Δ *lys2*Δ *ura3*Δ).

(4): From Page *et al*. (2003), *Genetics*, **163,** 875-894.
